# Generating patient-specific virtual tumor populations with reaction-diffusion models and molecular imaging data

**DOI:** 10.3934/mbe.2020341

**Published:** 2020-09-25

**Authors:** Nick Henscheid

**Affiliations:** Department of Medical Imaging, University of Arizona, 1501 N. Campbell, Tucson, AZ 85724, USA

**Keywords:** mathematical onoclogy, precision medicine, molecular imaging, virtual populations, virtual clinical trials, emission computed tomography

## Abstract

The use of mathematical tumor growth models coupled to noisy imaging data has been suggested as a possible component in the push towards precision medicine. We discuss the generation of population and patient-specific virtual populations in this context, providing in silico experiments to demonstrate how intra- and inter-patient heterogeneity can be estimated by applying rigorous statistical procedures to noisy molecular imaging data, and how the noise properties of such data can be analyzed to estimate uncertainties in predicted patient outcomes.

## 1. Introduction

Broadly speaking, mathematical oncology seeks to develop a collection of in silico models that accurately predict the growth and progression of malignant tumors and their response to treatment [1]. While the basic scientific goal is to develop a rigorous quantitative science of cancer [2], a more readily attainable goal is to use existing models and data to inform clinical practice in some substantive way [3]. One approach to clinical mathematical oncology, which we call mathematical model-based precision medicine, is to use mathematical models to make patient-specific prognostic and therapeutic predictions which are subsequently employed to direct treatment choices and predict patient outcomes. In our view, such predictions must be conditioned on patient data in order for the strategy to qualify as precision medicine [4-6].

Two essential features of oncology are heterogeneity and uncertainty. Heterogeneity can manifest as interpatient (between-patient), intrapatient (within-patient) and intratumor (within-tumor); see [7] for a more complete discussion of the clinical impact of heterogeneity in cancer and [8] for a review of the mathematical strategies to address heterogeneity. From a modeling perspective, heterogeneity implies a statistical approach where model parameters are treated as spatially distributed random variables with both population and patient-specific probability distributions [5]. Heterogeneity also highlights the issue of uncertainty: For a given patient, most if not all model parameters are unknown prior to the acquisition of patient-specific data, and even with such data, uncertainties may remain, since model parameters may only be partially identifiable and the data acquisition system may introduce additional irreducible noise. Image data provides a clear example of this issue, as imaging introduces information loss due to detector noise, low resolution and the possibility of null functions [9]. This uncertainty must be accounted for if model-based predictions are to be used reliably in the clinical context.

Once a relevant mathematical model has been selected, prediction takes place in silico, whereby a simulated patient is produced and Quantities-of-Interest (QoIs) are calculated. In this context, we say that the parameter values and resulting simulations correspond to a virtual patient. Owing to the heterogeneity and uncertainty issues discussed above, in both population and patient-specific applications of mathematical oncology it is desirable to generate suitably randomized Virtual Patient Populations (VPPs). If the VPP represents an untreated group, it is called a Virtual Control Population (VCP) [4, 10], while if the VPP represents a treatment group, it is called a Virtual Treatment Population (VTP). With appropriate VPPs, an in silico Virtual Clinical Trial (VCT) can be performed to assess treatment choices, again in either the population or patient-specific case. Population VPPs and VCTs can assist in making general prognosis and treatment recommendations or simulate a regulatory trial [11], while a patient-specific VPP and/or VCT can be used to assess uncertainty in a particular patient’s disease progression or treatment response [6, 12]. While population VCTs have found usage in device design and regulatory contexts [13], patient-specific VPPs and VCTs have seen limited usage.

In previous work [5, 6], we have highlighted the application of spatiotemporal random field modeling to address heterogeneity and uncertainty. In this work, we discuss specific VPP-based strategies towards the practical implementation of the framework presented in [5, 6]. Specifically, we describe a method for generating VCPs by pairing a spatiotemporal reaction-diffusion equation (RDE) model for avascular, pre-metastatic tumor growth with spatially inhomogenous random field coefficients. We discuss the RDE model in sections 2.1 and 2.2 and the random field models in section 2.3. Together, these models provide a solution of the ‘forward problem’ for VPP generation. Two important inverse problems then arise. In the first, we consider in sections 4.1 and 4.2 the problem of estimating the RDE coefficient fields for a specific patient from noisy molecular imaging data; proof-of-concept simulation results are given in sections 5.1 and 5.2. For the second inverse problem, we must estimate the statistical parameters that define the random field coefficient models from a collection of many patients’ data, hence solving the statistical calibration problem for virtual populations; we discuss this problem in section 4.3. Both maximum likelihood and Bayesian techniques are presented, and both require an accurate likelihood model connecting model parameters to available noisy data. To this end, in section 3 we discuss statistical models for molecular Emission Computed Tomography (ECT) data. We make this emphasis both because well-validated likelihood models for ECT are available [9], and because it is possible to model a direct connection between physiological processes such as tumor cell density and spatial growth rate, and certain emission imaging techniques and tracers.

Note that the results given in sections 5.1 and 5.2 are generated in a pure simulation setting, where both ground-truth tumors and image data are simulated. This allows for methodological comparisons with known ground truth and tight control of all nuisance parameters that influence the data. A summary of future work that extends these results is provided in section 6.

## 2. Tumor growth modeling with heterogeneity and uncertainty

In this section, we introduce the tumor growth model that we employ for the remainder of the paper. We emphasize that the models we present are simplifications of real tumor biology, which is complex and not yet fully understood. These models are intended to be more ‘clinically relevant’ [3] than biologically accurate, due to their relative simplicity and amenability to patient-specific inference from spatiotemporal imaging data. In section 2.2 we discuss the parameter-to-solution map, in section 2.3 we discuss random field models for the tumor growth model coefficients, and in section 2.4 discuss the notions of virtual populations and virtual clinical trials.

### 2.1. Reaction-diffusion tumor growth models

A wide variety of Partial Differential Equation (PDE) models have been employed to model spatiotemporal tumor growth, and the derivation and mathematical analysis of such models is adequately described elsewhere [14-21]. Here, we are interested in computational techniques for generating virtual patient populations, and thus consider a relatively simple PDE model (2.1). that has gained traction in the context of GlioBlastoma Multiforme (GBM). The randomization and statistical calibration techniques we present are independent of the tumor growth model, however, so the choice of Eq (2.1) is largely for illustration purposes.

We denote a spatiotemporal density of tumor cells by ***n*** = *n*(***x***, *t*), with ***x*** ∈ *V* being a *d*–dimensional position vector in the spatial domain *V* and 0 ≤ *t* ≤ *T*, where *T* is the maximum number of days simulated. The units of ***n*** are cells per unit volume, and we take (***x***, *t*) to have units of cm and days, respectively. For in vivo tumors *d* = 3, though for simulation purposes and modeling of in vitro and certain in vivo experimental setups we also consider spatial domains in *d* = 2 as a reasonable approximation. The general reaction-diffusion equation for ***n*** is

(2.1) {∂n∂t=∇⋅(D∇n)+G(n)(x,t)∈V×[0,T]n(x,0)=n0(x)v^⋅∇n=0boundary conditions}

The spatially varying scalar diffusion coefficient ***D*** = *D*(***x***) describes the rate of apparent cell diffusion, while the growth function G(n) describes the growth and competition between the tumor and its environment. The boundary condition is taken to be the no-flux (Neumann) condition $\hat{v}\cdot \nabla n=0$, where $\hat{v}$ is the outward boundary normal of the domain *V*. Note that a treatment function T(n) can be added to Eq (2.1) to model an intervention such as chemotherapy, radiotherapy or surgical resection, but we postpone discussion of such models to a future work.

While in the scalar tumor growth context the Gompertz function G(n)=−ρnln(n∕κ) has gained plausible mechanistic support via the notion of quiescence [22], to our knowledge the spatial RDE growth function is largely a phenomenological choice. The Fisher-KPP growth function is frequently selected in the GBM context, and is the one we will use in this paper:

(2.2) $$G(n)=\rho (x)n(x,t)\left(1-\frac{n(x,t)}{\kappa (x)}\right)$$

The growth *ρ*(***x***) and carrying capacity *κ*(***x***) functions, which have respective units of cells per day and cells, will be discussed further below.

### 2.2. Patient-specific parameters and the parameter-to-solution map

Note that in Eqs (2.1) and (2.2), several additional parameter functions are required to fully specify the solution to the PDE. These include the diffusion coefficient *D*(***x***), the initial cell density *n*_0_(***x***), the growth *ρ*(***x***) and carrying capacity *κ*(***x***). These parameter functions are certainly patient-specific, since they correspond to local physiology and nutrient concentrations. For notational simplicity, we will use the symbol ***β*** to denote the collection of all parameters necessary to specify a solution to Eq (2.1). In the context of this paper, a virtual patient then corresponds to a particular choice of ***β***, which we will indicate by saying that patient *j* has parameter ***β***_*j*_. We will assume for the remainder of this article the initial condition is given by a Gaussian

(2.3) $$n_{0}(x)=A\;\text{exp}\left(\frac{1}{2\sigma^{2}}-\|x-x_{0}{\|}^{2}\right)$$

with ***x***_0_ = [0.5, 0.5]^*T*^, *A* = 5/2*πσ*^2^ and *σ*^2^ = 1*e*−4; this represents an initial collection of 5 tumor cells, well-localized around the center of our computational domain. The collection of all remaining unknown parameter fields is thus ***β*** = (***D***, ***ρ***, ***κ***). To avoid pathological situations, we also assume the technical condition that β∈B, where B is a set of parameter functions selected to guarantee that a positive, bounded solution *n*(***x***, *t*) of Eq (2.1) exists for all β∈B and for 0 ≤ *t* ≤ *T*. Selecting such a B requires careful technical analysis of Eq (2.1); see [23].

While we assume that each patient corresponds to a unique set of parameters ***β*** = ***β***_*j*_, it is likely that ***β***_*j*_ is effectively unknown or uncertain for a given patient. To address this, we treat ***β*** as a function space-valued random vector in both the population and patient-specific cases, and provide corresponding random field models in section 2.3 below. In a simulation setting, we must choose a computational representation for ***β***_*j*_, which we indicate with a superscript *v* for ‘virtual’. So, $\beta_{j}^{\left(v\right)}$ is a virtual (i.e., computational) sample corresponding to a sample for ***β***.

Assuming Eq (2.1) is classically well-posed for every (or almost every) β∈B, we can construct the deterministic parameter-to-solution map

(2.4) $$n_{j}^{\left(model\right)}=F(\beta_{j})$$

where $n_{j}^{\left(model\right)}=n_{j}^{\left(model\right)}$ (***x***, *t*) solves Eq (2.1) with the parameter vector ***β*** = ***β***_*j*_, corresponding to patient *j*. In a simulation setting, the forward map (2.4) is implemented via a numerical differential equation solver. We have employed a finite difference method-of-lines approach with a five-point spatial stencil and Runge-Kutta time stepper, as discussed in [24]. The result is an approximate solution to Eq (2.1), where the virtual coefficient sample $\beta_{j}^{\left(v\right)}$ is processed through computational algorithm $F^{\left(v\right)}$ to produce the virtual tumor cell density $n_{j}^{\left(v\right)}$:

(2.5) $$n_{j}^{\left(v\right)}=F^{\left(v\right)}\left(\beta_{j}^{\left(v\right)}\right)$$

**Figure 1.**
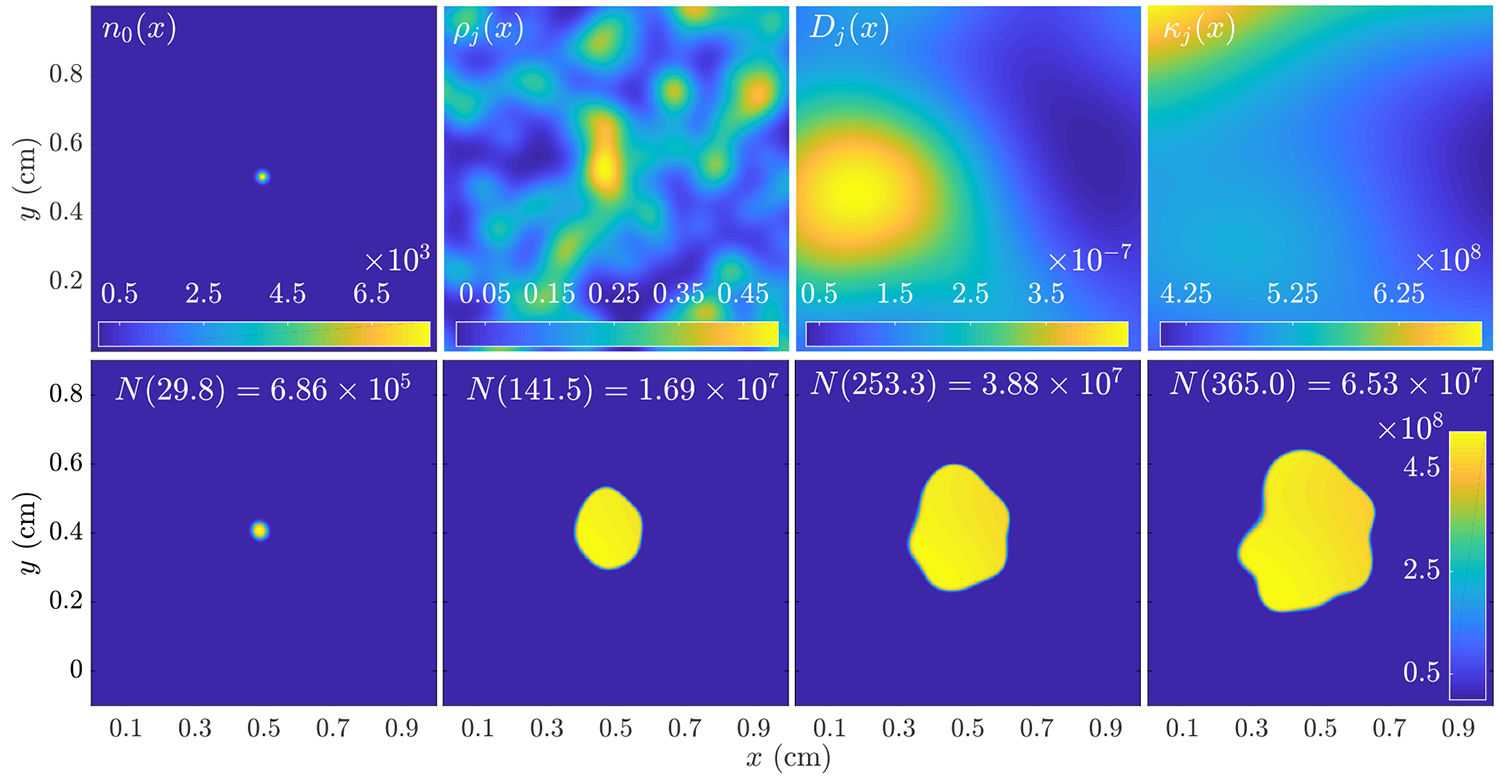
A realization of the tumor growth model (2.1) with Fisher-KPP growth term (2.2) (bottom row). The time-independent random field coefficients (top row, frames 2–4) are draws from ***ρ*** ~ *LB*(200, 0.1, 2e–3), ***D*** ~ *LB*(20, 1e–7, 0.04) and ***κ*** ~ *LB*(100, 5e7, 0.1), as defined in section 2.3. The tumor burden *N*(*t*), defined in Eq (2.13), is shown for each time point. The initial condition *n*_0_(***x***) is shown top left.

The choice of discretization specifies the format for the virtual cell density $n_{j}^{\left(v\right)}$; with our finite difference scheme, we can assume that $n_{j}^{\left(v\right)}$ takes the form of an *N*_*x*_ × *N*_*y*_ × *N*_*t*_ voxel array (for *d* = 2). The specification of $\beta_{j}^{\left(v\right)}$ is discussed below; we will assume a voxel-free form (Eq (2.8) below) that can be evaluated on an arbitrary grid when needed. Note that the virtual cell density will differ both from a patient’s true cell density and from a measured cell density by discretzation, model and measurement errors; see [8] for a discussion of these errors. A demonstration of our scheme implementing (2.5) in *d* = 2 is shown in Figure 1.

In the next section, we will introduce random field models that allow Eqs (2.4) and (2.5) to be used to generate virtual tumor populations. Note briefly that we have made assumptions that allow us to analyze and simulate Eq (2.1) as a Random PDE (RPDE) model, that is as a classically well-posed PDE whose coefficients are randomized. Note that this is in contrast to a Stochastic PDE (SPDE) model, which requires more advanced analysis and simulation techniques. See e.g., [5] for discussion of a technique based on characteristic functionals (introduced briefly below), and [25-27] for further discussion of the distinction between SPDE and RPDE.

### 2.3. Random field models for RDE coefficients

As discussed in the introduction, any mathematical model that is employed in a clinical precision medicine context must account for the possibility of heterogeneity and uncertainty by assuming that model parameters are sampled from a probability distribution. Since the model parameters that specify Eqs (2.1) and (2.2) are spatial functions, we employ the theory of random fields. We will avoid technical discussions, sufficing to say that one can treat ***β*** as a function space-valued random vector, written formally as β:Ω→B, where (Ω, F, P) is a probability space and B is a set of functions mentioned previously, selected to guarantee the existence of the forward map (2.4). We assume that B⊂X, where X is a Hilbert space with inner product (***β***, ***φ***)X, taken to be the *L*^2^(*V*) inner product for vector-valued functions. In the most general setting, the complete statistics of a second-order random process are fully specified by its marginal distributions (through the Kolmogorov construction), its abstract probability distribution $P_{\beta }$, or its characteristic functional: [5, 9, 28, 29]

(2.6) $$\Psi_{\beta }(\varphi )=E\left[\text{exp}\left(i(\beta ,\varphi {)}_{X}\right)\right].$$

Frequently, a process is completely specified by its first few moments e.g., its mean function $\bar{\beta }(x)=E[\beta ]$ and its covariance function $k(x,x^{\prime })=E[(\beta (x)-\bar{\beta }(x))(\beta (x^{\prime })-\bar{\beta }(x^{\prime }){)}^{T}]$. While its use requires functional calculus, the characteristic functional (2.6) is typically a compact and efficient way to describe the complete statistics of a random field, and its use in the biological context is a topic of current investigation [5]. Refer to the recent books [27] and [30] for a complete discussion of the theory of Hilbert space-valued random vectors.

In this work, we will simulate random fields by first assuming a convenient functional form for the realizations of the process, then randomizing the corresponding (finitely many) parameters to achieve desired statistics. This method allows us to guarantee that realizations of ***β*** satisfy conditions for Eq (2.1) to be well-posed, and allows for straightforward simulations using standard code packages. In particular, the random field models we use will take the form of a randomized synthesis map, which for a scalar-valued function *φ*_*j*_(***x***) takes the form:

(2.7) $$\varphi_{j}=\Phi (\theta_{j})$$

where $\Phi (\cdot )\;:R^{N}\to X$ is a synthesis map that maps a (finite-dimensional) patient-specific parameter ***θ***_*j*_ to the function $\varphi_{j}^{\left(v\right)}(x)$. By selecting a probability distribution $P(\theta_{j}|\theta_{p})$ that depends on the population parameter $\theta_{p}\in \Theta \subset R^{p}$, the synthesis map (2.7) produces a random field. In sections 4.1 and 4.2, we discuss ways to estimate a particular ***θ***_*j*_ from image data, while in section 4.3 we discuss ways to estimate the population parameter ***θ***_*p*_ from a collection of images.

Because the coefficients in the RDE (2.1) must be non-negative and bounded to be physiologically realistic, it is advantageous to begin with a random field model that always produces non-negative, bounded realizations. For our simulations, we employ ‘lumpy-type’ random field models [31, 32], a realization of which has the functional form

(2.8) $$\varphi (x)=\sum_{l=1}^{L_{max}}b_{l}\ell (x-x_{l};\gamma ).$$

If the lump function *ℓ*(***x***; ***γ***) and the lump amplitudes *b*_*l*_ are non-negative and bounded, and the number of lumps is finite, then Eq (2.8) is guaranteed to produce non-negative and bounded realizations. For simplicity, we use isotropic unnormalized Gaussian lumps:

(2.9) $$\ell (x;\sigma^{2})=\text{exp}\left(-\frac{1}{2\sigma^{2}}\|x{\|}^{2}\right)$$

Random fields with this choice of lump function were proposed to model soft tissues with smoothly varying characteristics [31], while more sophisticated choices of *ℓ*(***x***), such as the clustered lump technique [32], can model more complex tissues such as breast or brain tissue. Ultimately, the choice of *ℓ*(***x***) and its shape parameter ***γ*** requires a statistical calibration step such as that discussed in section 4.3.

We randomize Eq (2.8) as follows. We first select a lump shape parameter $\gamma =\sigma^{2}=\sigma_{0}^{2}$, a constant lump amplitude *b*_0_, and a mean number of lumps $\bar{L}\ll L_{max}$. We then draw $L∼\text{Poi}(\bar{L})$, and set *b*_*ℓ*_ = *b*_0_ for 1 ≤ *ℓ* ≤ *L*, and *b*_*ℓ*_ = 0 otherwise. Lastly, we draw ***x***_1_ … , ***x***_*L*_, I.I.D. uniform in the domain *V* and form the sum (2.8). Note that the resulting random field realization can be written in terms of a nonlinear synthesis map (2.7) with *N* = (*d* + 1)*L*_*max*_. We use the notation $LB(\bar{L},b_{0},\sigma_{0}^{2})$ to indicate a lumpy-type field with mean number of lumps $\bar{L}$, amplitude *b*_0_ and isotropic lump variance $\sigma_{0}^{2}$. A demonstration of the behavior of lumpy-type random fields of the form given in Eq (2.8) is shown in Figure 1, top row.

We reiterate that a single realization of the random field specified in Eq (2.8) is fully specified by the parameters $\sigma_{0}^{2}$, *b*_0_ and the lump center list [***x***_*l*_], while the complete statistics of the random field are fully specified by $\bar{L}$, $\sigma_{0}^{2}$ and *b*_0_. This distinction is important when we discuss parameter estimation problems in sections 4.1-4.3.

### 2.4. Virtual populations and quantitative biomarkers

Suppose that $q\in R^{n}$ is a low-dimensional vector quantifying patient status or prognosis, for instance (informally) ***q*** = [metric of tumor burden, metric of normal tissue function]. In the model-based precision medicine context, such a vector is generally called a quantitative biomarker [6, 33, 34]. The purpose of a virtual patient population, and more generally a virtual clinical trial, is to predict the distribution of ***q*** for either a control or treatment group. We will assume for convenience that q=q∈R is a scalar functional of ***β*** and ***n***:

(2.10) q=M(β,n)

Given a virtual population of simulated parameters and tumors, $\left(\beta_{1}^{\left(v\right)},n_{1}^{\left(v\right)}\right)$, … , $\left(\beta_{J}^{\left(v\right)},n_{J}^{\left(v\right)}\right)$, we estimate the biomarker *q* for each virtual patient using a method $M^{\left(v\right)}$, resulting in a vector

(2.11) $$Q^{\left(v\right)}=\left[M^{\left(v\right)}\left(\beta_{1}^{\left(v\right)},n_{1}^{\left(v\right)}\right),\ldots ,M^{\left(v\right)}\left(\beta_{J}^{\left(v\right)},n_{J}^{\left(v\right)}\right)\right]\in R^{J}$$

If ***Q***^(*v*)^ is generated from a population distribution, that is, the random vector ***β*** models inter-patient heterogeneity, then we say that ***Q***^(*v*)^ is a *population* virtual biomarker sample. If ***β*** models patient-specific uncertainties, for instance if ***β*** is sampled from the conditional random vector ***β***_*j*_∣***g***_*j*_ where ***g***_*j*_ is some patient-specific data, then we say that ***Q***^(*v*)^ is a patient-specific virtual biomarker sample. Statistical properties of *q* such as its mean, standard deviation and distribution can be estimated from ***Q***^(*v*)^. By generating virtual control and treatment groups, Eq (2.11) can be used to simulate a clinical trial. If the VPP employed in Eq (2.11) represents a treatment group, decisions regarding the intervention can be made based on these statistics. For instance, a rigorous decision-theoretic approach to patient-specific treatment selection would define an additional function *U*(***q***) which measures the utility of achieving the biomarker ***q***(***τ***) via the intervention ***τ***. Since ***q*** is a random vector, the typical approach is to maximize the expected utility, that is, we seek the intervention τ* which solves

(2.12) $$\tau^{*}=\underset{\tau }{\text{arg}\;\max}E\left[U(q(\tau ))\right]$$

While numerous patient-specific therapy optimization schemes have been suggested [35], to our knowledge a scheme based on Eq (2.12) has not seen practical implementation in this context. The VPP methodologies presented here may prove applicable to a future application of Eq (2.12), since the expected value in Eq (2.12) can be approximated via Eq (2.11).

An example of a quantity-of-interest that we will use to illustrate our methods is the tumor burden, which is the total number of tumor cells as calculated from *n*(***x***, *t*):

(2.13) $$N(t)=\int_{V}n(x,t)\;dx$$

**Figure 2.**
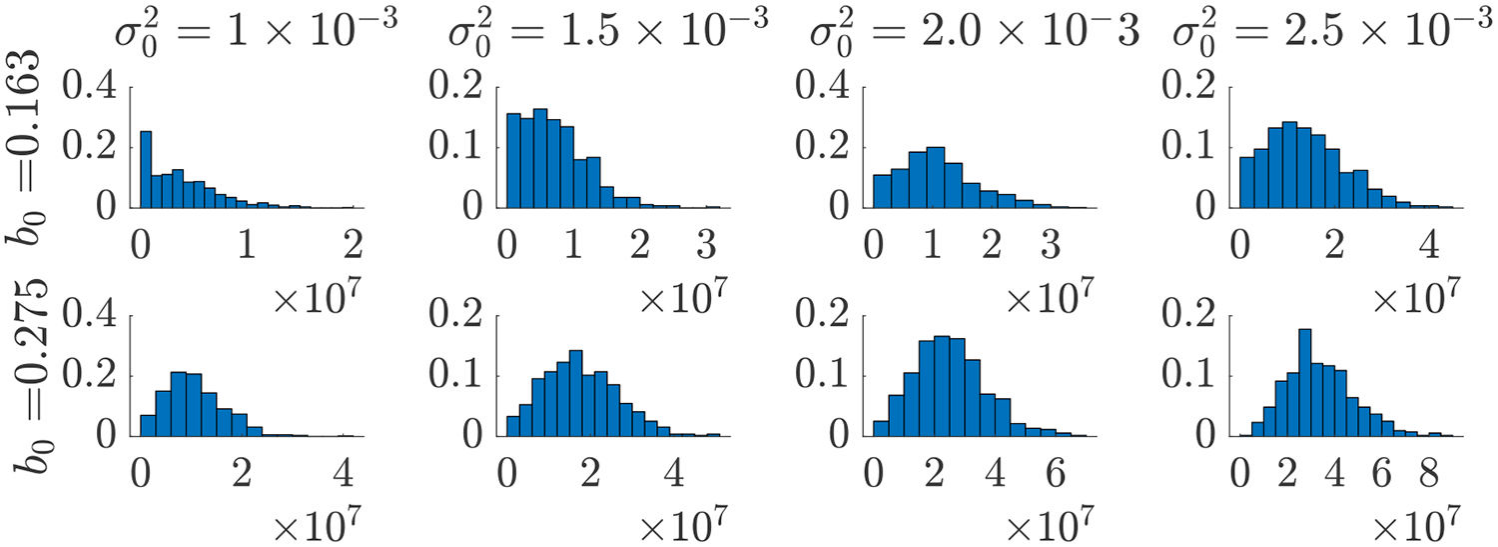
Demonstration of the influence of the random field parameters ***θ***_*p*_ on the distribution of the biomarker (2.13). The parameters (*b*_0_, $\sigma_{0}^{2}$) controlling the statistics of $\rho (x)∼LB(200,b_{0},\sigma_{0}^{2})$ are changed, and for each pair, a virtual biomarker sample of size *J* = 512 is computed with $q=M^{\left(v\right)}(n^{\left(v\right)})=N(100)$. Relative frequency histograms are displayed for each virtual biomarker sample.

If the goal is to predict *N*(*t*) for some *t* in the future, a VPP allows one to assess the uncertainty in this value as a result of uncertainty in the patient-specific parameter ***β***_*j*_ being ‘propagated’ through the models F and M [25, 30]. An illustration of uncertainty propagation is demonstrated in Figure 2, where several choices of the population parameter ***θ***_*p*_ are chosen and the resulting (estimated) probability distribution of *N*(100) is shown. Further examples are given in section 5 below. Another possible QoI, the integrated log-kill, is similar to Eq (2.13) but with the integrand *n*(***x***, *t*) replaced by ln(*n*(***x***, *t*)/*n*_0_(***x***)). This metric is derived from a mass action drug effect and is applicable in chemotherapy evaluation [5]. Note that we do not discuss the important topic of exactly which scalar QoIs have real clinical impact in terms of patient outcomes; refer to e.g., [33, 34].

## 3. Molecular emission imaging data

In a pure simulation setting, a virtual biomarker sample of the form shown in Eq (2.11) can be computed without reference to any particular patient-specific data, so long as the corresponding probability distributions are well-calibrated. However, the model-based precision medicine context requires that patient-specific data be used to create individualized VPPs, hence making uncertainty-informed decisions about patient *j* possible. We will focus on the case of molecular Emission Computed Tomography (ECT) data, for reasons discussed below.

### 3.1. Preclinical and clinical imaging modalities

In the clinic, image data typically takes the form of reconstructed tomographic imaging studies such as MRI, CT, or ^18^F-FDG Positron Emission Tomography (PET). Note that while it can be difficult to precisely relate these standard tomographic data to either ***n***_*j*_ or ***β***_*j*_, it is nonetheless possible to postulate such relationships and thereby extract useful information from these images. An example of such an approach is the usage of Apparent Diffusion Coefficient (ADC) maps, generated using Diffusion-Weighted MRI (DWMRI), to estimate ***n***_*j*_ through the application of a model which relates tissue cellularity to ADC [3, 36, 37]. The basic difficulty in such efforts is that malignancy is not directly measured by any of these standard techniques: For example, ADC maps estimate the apparent diffusion coefficient of water molecules and CT measures X-Ray attenuation, neither of which is uniquely altered by the presence of a malignant tumor. While increased ^18^F-FDG uptake implies increased cellular metabolism along the glycolytic pathway, a byproduct of malignant growth, this effect is not unique to malignant cells, since benign tumors can also display an increased metabolic rate [38].

In this article we consider a wide class of imaging techniques known as Emission Computed Tomography (ECT), broadly defined as any imaging modality (such as PET) which detects a molecular tracer distribution via optical or nuclear detection techniques. We choose to focus on ECT data because the wide variety of available tracers allows for more direct modeling relating these data to tumor cell density ***n***_*j*_ and the RDE coefficient fields ***β***_*j*_. As an example, genetically engineered cancer cells can be made to express Green Fluorescent Protein (GFP), which acts as a photon activity distribution when excited [39], and thus ECT, typically in the form of intravital microscopy (e.g., window chambers [40]) can be used to image ***n***_*j*_ directly in vivo, at least in the small animal setting. Meanwhile, the growth factor ***ρ***_*j*_ can potentially be measured using the PET tracer ^18^F-FLT, which is a radioactive version of the molecule thymadine used in DNA synthesis [41, 42]. Ex vivo, the Ki-67 immunohistochemistry stain would typically be employed to measure proliferation, but this requires either a biopsy or sacrifice of the animal. Current progress in small animal imaging is working towards producing in vivo imaging agents for common immunohistochemistries, but to our knowledge an in vivo Ki-67 analog is not available. The other advantages of ECT are the possibility to perform polyscopic imaging (that is, measurement of several distinct physiological processes simultaneously) [43], and the wealth of literature on the statistics of raw and reconstructed ECT data [9].

To employ the statistical estimation strategies discussed in section 4, we require statistical models for ECT data. We emphasize the case of raw, un-reconstructed ECT data because the statistics are better understood: Most reconstructed images produced in the clinic are generated by a proprietary ‘black box’ method provided by the imaging system manufacturer. Our framework can still be applied to such data by carefully modifying the H operator defined in Eq (3.3) to account for the reconstruction step, however it can be difficult to calibrate an appropriate noise model for likelihood-based estimation. If the MLEM algorithm is known to have been employed for reconstruction, see [44] for a discussion of the resulting noise characteristics.

### 3.2. Statistics of ECT data

In an ECT system, a chemical tracer is engineered to emit some measurable particle, such as photons, charged particles or nuclear decay products [45]. A tracer corresponds to a particle activity distribution *f*_*j*_(***x***, *t*), having units of particles emitted per unit volume per unit time. For the case of GFP imaging discussed above, a reasonable model is $f_{j}^{\left(GFP\right)}(x,t)\propto n_{j}(x,t)$, that is, the photon activity is proportional to the actual tumor cell density, with constant of proportionality related to the photon yield per cell (i.e., the number of photons detected, on average, per cell). Similarly, we can assume $f_{\;j}^{{(}^{18}F\text{-}\mathrm{FLT})}\propto \rho_{j}$, the growth factor. The energy produced by *f*_*j*_(***x***, *t*) propagates through the tissue medium and is detected by specialized ECT imaging hardware, which can range from optical CCD and CMOS detectors to scintillation-type detectors for high-energy particles. The statistics of raw ECT data are discussed at length elsewhere [9, 46], so we summarize by noting that two data formats are commonly available, known as binned-mode data and particle-processed data. Because the presentation of particle-processing requires a more technical discussion of imaging detectors, we only consider binned-mode data in this work; see e.g., [45-47] for a discussion of particle processing. In a future work we will address the usage of particle processing data in mathematical oncology.

Binned-mode ECT data arises when detected particles are sorted into spatial bins corresponding to a discretization of the detector. These spatial bins need not correspond to physical detector pixels, which many ECT imaging detectors do not have. The physics-based statistics of particle counting nearly always lead to a Poisson data model for particle count data, which can be written in terms of a noisy linear functional of the activity ***f***_*j*_ as follows. Suppose that the imaging system has *M* detector bins, with data for patient *j* labeled *g*_*j*1_, … , *g*_*jM*_. The statistical model for binned-mode ECT data is that ***g***_*j*_ is a Poisson random vector with mean

(3.1) $${\bar{g}}_{jm}=\int_{0}^{T}\int_{V}h_{m}(x,t)f_{j}(x,t)\;dxdt$$

where *h*_*m*_(***x***, *t*) is called the *m*-th sensitivity function of the imaging system (assumed to be fixed for all patients), *f*_*j*_(***x***, *t*) is the particle activity distribution corresponding to the tracer administered to patient *j*, and *T* is the total imaging time. For tomographic systems, *h*_*m*_(***x***, *t*) must account for both the propagation within the patient, typically following a Radiative Transport Equation (RTE), as well as the geometry and blur characteristics of the imaging detector [6, 9]. For this work, we will consider a simplified imaging geometry, intended to model intravital microscopy techniques such as mouse window chambers, where optical and/or gamma-ray images are collected with a planar detector above a pseudo-2D activity distribution. To a reasonable approximation, a Gaussian blur model

(3.2) $$h_{m}(x,t;A,\sigma_{blur}^{2})=\frac{A}{2\pi \sigma_{blur}^{2}}\;\text{exp}\left(-\|x-x_{m}{\|}^{2}∕2\sigma_{blur}^{2}\right)$$

is then appropriate. This blur accounts for both imaging elements such as lenses and collimators as well as position estimation blur [9, 46]. More sophisticated imaging system models, for instance 3D tomographic systems, will be discussed in a future work (see section 6).

Expressing Eq (3.1) as a linear continuous-to-discrete operator $H\;:X\to R^{M}$ allows us to write a binned-mode system model succinctly as

(3.3) $$g_{j}={\bar{g}}_{j}+\eta_{j}=Hf_{j}+\eta_{j},$$

where ${\bar{g}}_{j}=[{\bar{g}}_{j1},\ldots ,{\bar{g}}_{jM}{]}^{{}_{T}}=Hf_{j}\in R^{M}$ is defined via Eq (3.1). Considering ***f***_*j*_ as a parameter, the probability distribution for the count vector ***g***_*j*_ is thus

(3.4) $$P(g_{j}|f_{j})=\prod_{m=1}^{M}\frac{\text{exp}(-{\bar{g}}_{jm}){\bar{g}}_{jm}^{g_{jm}}}{g_{jm}!}=\prod_{m=1}^{M}\frac{\text{exp}\left(-(Hf_{j}{)}_{m}\right)(Hf_{j}{)}_{m}^{g_{jm}}}{g_{jm}!}$$

If ***f***_*j*_ has been parameterized using a finite-dimensional parameter ***θ***_*j*_, for instance via a synthesis map of the form shown in Eq (2.8), we can define a nonlinear function $H\;:R^{N}\to R^{M}$, where for image reconstruction typically *N* < *M*, as follows:

(3.5) $$H(\theta_{j})=H\Phi (\theta_{j})$$

This function computes the mean image data ${\bar{g}}_{j}$ for the synthesized object ***f***_*j*_ = **Φ**(***θ**_j_*), and is implemented computationally via numerical quadrature methods applied to Eq (3.1). The component functions of ***H*** are denoted $H_{m}(\theta_{j})\;:R^{N}\to R$. Note that if the synthesis method is nonlinear, as in Eq (2.8), then ***H*** is a nonlinear function of the vector ***θ***_*j*_, while if **Φ** is linear, then ***H*** is linear. In either case, we can write a probability distribution for ***g***_*j*_∣***θ***_*j*_ as follows:

(3.6) $$P(g_{j}|\theta_{j})=\prod_{m=1}^{M}\frac{\text{exp}\left(-H_{m}(\theta_{j})\right)H_{m}(\theta_{j}{)}^{g_{jm}}}{g_{jm}!}$$

We will make use of Eqs (3.4)-(3.6) for likelihood-based estimation methods in section 4 below.

## 4. Statistical estimation and inversion techniques

Assume that for patient *j*, ECT data $g_{j}\in R^{M}$ has been collected, where ***g***_*j*_ can either be monoscopic (a single imaging study) or polyscopic (consisting of multiple imaging studies, corresponding to multiple tracers). In the previous section, we provided statistical models for ***g***_*j*_ that relate it to the activity distribution ***f***_*j*_ via Eq (3.3). As discussed there, ECT tracers allow ***f***_*j*_ to be directly related to ***n***_*j*_ and ***ρ***_*j*_. In this section, we discuss statistical techniques for estimating ***f***_*j*_ (hence ***n***_*j*_ and ***ρ***_*j*_) from ***g***_*j*_. In section 4.1, we discuss point estimation techniques for an individual patient, where a single estimate of ***f***_*j*_ is produced, then in section 4.2 we discuss patient-specific Bayesian strategies where the solution consists of samples from a posterior distribution. In section 4.3, we discuss methods for estimating a population parameter ***θ***_*p*_ from a database ***G*** = [***g***_1_, … , ***g***_*J*_] of images collected for ***J*** patients.

### 4.1. Patient-specific maximum likelihood parameter estimation

Given data $g_{j}\in R^{M}$ for patient *j*, we consider the estimation of the underlying activity distribution ***f***_*j*_ using the imaging system model $g_{j}=Hf_{j}+\eta_{j}$, given in Eq (3.3). To approach this infinite-dimensional inverse problem, we select a finite-dimensional parameterization ***f***_*j*_ = **Φ**(***θ***_*j*_), where $\theta_{j}\in R^{N}$, and consider the problem of estimating ***θ***_*j*_ from ***g***_*j*_, that is, solving the inverse problem ***g***_*j*_ = ***H***(***θ***_*j*_)+ ***η***_*j*_. Note that moving from ***f***_*j*_ to ***θ***_*j*_ might introduce an approximation error, owing to the fact that ***f***_*j*_ need not be expressible in same form as the synthesis used for the reconstruction; in ECT imaging, ***f***_*j*_ is typically quite smooth, so this approximation error is typically insignificant when compared to the influence of detector resolution and noise. While typical image reconstruction algorithms assume a voxel-based parameterization of ***f***_*j*_, we elect to use a lumpy-type synthesis of the form given in Eq (2.8). As discussed, the activity distribution can correspond to the cell density ***n***_*j*_, or the growth factor *ρ*_*j*_(***x***). We denote a generic estimate of ***θ***_*j*_ produced from ***g***_*j*_ by a hat, i.e., ${\hat{\theta }}_{j}$ is an estimate of ***θ***_*j*_ produced from the data ***g***_*j*_. While a wide array of strategies exist to solve this inverse problem, we consider only likelihood-based methodologies.

Recall from section 3.2 that the probability distribution for binned-mode ECT data is Poisson. For an activity distribution ***f***_*j*_ parameterized using the synthesis map **Φ**, i.e. ***f***_*j*_ = ***f***_*j*_(***θ***_*j*_), we thus have a likelihood function

(4.1) $$L(\theta_{j}|g_{j})=P(g_{j}|\theta_{j})=P(g_{j}|f_{j}(\theta_{j}))=P(g_{j}|\Phi (\theta_{j}))=\prod_{m=1}^{M}\frac{\text{exp}\left(-H_{m}(\theta_{j})\right)H_{m}(\theta_{j}{)}^{g_{jm}}}{g_{jm}!}$$

where *P*(***g***_*j*_∣***f***_*j*_) is the Poisson distribution given in Eq (3.4), and *H*_*m*_(***θ***_*j*_) are the components of the function defined in Eq (3.5). Note the abuse of notation in Eq (4.1): The two functions *P*(***g***_*j*_∣***θ***_*j*_) and *P*(***g***_*j*_∣***f***_*j*_) have different parameter types, but we take Eq (4.1) to define the former in terms of the latter. Taking its logarithm (and ignoring the ***θ***_*j*_-independent constant) results in the log-likelihood function

(4.2) $$\ell (\theta_{j}|g_{j})=\text{ln}(L(\theta_{j}|g_{j}))=\sum_{m=1}^{M}\left[g_{jm}\;\text{ln}({\bar{g}}_{jm})-{\bar{g}}_{jm}\right]=\sum_{m=1}^{M}\left[g_{jm}\;\text{ln}(H_{m}(\theta_{j}))-H_{m}(\theta_{j})\right]$$

We now define the Maximum Likelihood Estimate (MLE) as the maximum of either Eq (4.1) or Eq (4.2) over the set **Θ** of allowed parameters:

(4.3) $${\hat{\theta }}_{j}^{\left(ML\right)}=\underset{\theta \in \Theta }{\text{arg}\;\max}L(\theta |g_{j})=\underset{\theta \in \Theta }{\text{arg}\;\max}\ell (\theta |g_{j})=\underset{\theta \in \Theta }{\text{arg}\;\text{min}}\left[-\ell (\theta |g_{j})\right]$$

A variety of optimization algorithms exist to compute ${\hat{\theta }}_{j}^{\left(ML\right)}$, including the Expectation Maximization (EM) algorithm and Newton-type methods [48, 49]. If the imaging operator ***H***(***θ***_*j*_) is linear, the EM algorithm applied to Poisson data leads to an iterative method known in the imaging community as the MLEM algorithm. Starting from ${\hat{\theta }}_{j}^{\left(0\right)}$, the MLEM algorithm performs the iterative update

(4.4) $${\hat{\theta }}_{jn}^{\left(k+1\right)}=\frac{{\hat{\theta }}_{jn}^{\left(k\right)}}{s_{n}}\sum_{m=1}^{M}H_{mn}\frac{g_{m}}{{\left(H{\hat{\theta }}_{j}^{\left(k\right)}\right)}_{m}},\;\;s_{n}=\sum_{m=1}^{M}H_{mn}$$

**Figure 3.**
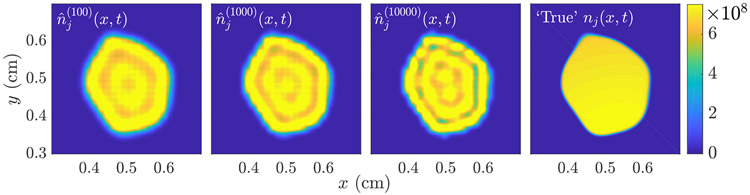
Illustration of the MLEM image reconstruction algorithm (4.4) for *k* = 10^2^, 10^3^ and 10^4^ iterations. The true *n*_*j*_(***x***, *t*) shown on the right. The visible artifacts are due to the fact that H has a nontrivial null space which is not accounted for in the MLE (4.3). Regularization strategies can alleviate this, though we have found that for the task of predicting tumor burden *N*(*t*), these artifacts have minimal impact.

We will employ the method (4.4) to compute some MLEs for linear imaging models in section 5, while for nonlinear models we use the Matlab algorithm fmincon, which is a based on an interior point method with BFGS quasi-Newton steps [50]. A demonstration of algorithm (4.4) applied to a reconstruction of *n*_*j*_(***x***, *t*) from image data of the form (3.1) is shown in Figure 3. Note that the reconstruction displays visual artifacts due to the function ***H***(***θ***) having a nontrivial null space. We have found that these artifacts have minimal impact on the task at hand, which is to predict the progression of *n*(***x***, *t*) and *N*(*t*). Future work will analyze in more detail the question of task-based image quality, as defined in [9, 51], in the mathematical oncology context.

We note briefly that under certain technical conditions, an MLE is asymptotically consistent and efficient, meaning for well-specified likelihood models, the MLE converges to the true parameter and attains the Cramér-Rao lower bound. Asymptotically, the distribution of the estimate ${\hat{\theta }}_{j}^{\left(ML\right)}$ (as a random variable depending on the data ***g***_*j*_) converges to a normal with covariance given by the inverse of the Fisher information [9]:

(4.5) $$\sqrt{M}\left({\hat{\theta }}_{j}^{\left(ML\right)}-\theta_{j}\right)\to N(0,F^{-1}),\;\;F_{nn^{\prime }}=-E_{g_{j}}\left(\frac{\partial^{2}\ell (\theta_{j}|g_{jm})}{\partial \theta_{jn}\partial \theta_{jn^{\prime }}}\right)$$

The Fisher information matrix ***F*** = ***F***(***θ***_*j*_) can be used to design more efficient measurement systems by ‘tuning’ the design parameters of the system to modify the likelihood in a desirable manner [52]. Note also that the Fisher information matrix can be evaluated ‘off-line’, by computing the expected values in Eq (4.5), which allows one to analyze the uncertainty in the MLE in a frequentist setting. While ***F*** depends on the true ***θ***_*j*_, for many ECT systems this dependence is minimal (i.e., the Fisher information matrix is nearly constant with respect to ***θ***_*j*_), which allows one to compute a single (system-dependent) ***F*** to be used for all MLEs derived from the system. One can also estimate confidence intervals for scalar quantities of interest derived from estimates of ***θ***_*j*_ using transformation rules for the Fisher information matrix [53].

To use the MLE procedure to generate a patient-specific virtual population, we assume that $g_{j}=Hf_{j}$, where either ***f***_*j*_ = *n_j_*(***x***, *t*_0_), ***f**_j_* = ***ρ***_*j*_, or ***f***_*j*_ = [*n_j_*(***x***, *t*_0_), *ρ*_*j*_(***x***)]^*T*^, that is, we image either the cell density at a single time point, or the growth factor, or both the cell density and the growth factor. From this data, we take the following steps:

- Use MLE to obtain ${\hat{\theta }}_{j}^{\left(n\right)}$ (and ${\hat{\theta }}_{j}^{\left(\rho \right)}$, if measured) from which we synthesize ${\hat{n}}_{j}$ (and ${\hat{\rho }}_{j}$, if measured).
- Randomize the remaining parameters ***D***, ***κ*** (and ***ρ***, if not measured) using a well-calibrated random field model such as Eq (2.8), resulting in $D_{j}^{\left(1\right)}$, … , $D_{j}^{\left(J\right)}$, $\kappa_{j}^{\left(1\right)}$, … , $\kappa_{j}^{\left(J\right)}$ (and $\rho_{j}^{\left(1\right)}$, … , $\rho_{j}^{\left(J\right)}$, if required).
- Generate a patient-specific virtual population by using ${\hat{n}}_{j}$ as the initial condition, either ${\hat{\rho }}_{j}$ or $\rho_{j}^{\left(1\right)}$, … , $\rho_{j}^{\left(J\right)}$ for the growth factor, and $D_{j}^{\left(1\right)}$, … , $D_{j}^{\left(J\right)}$, $\kappa_{j}^{\left(1\right)}$, … , $\kappa_{j}^{\left(J\right)}$ for the diffusion coefficient and carrying capacity, and solving the forward problem (2.5) for each sample.
- With the resulting VPP, compute any desired quantitative biomarkers as in Eq (2.11).

Note that in the above procedure, we make no attempt to estimate ***D*** and ***κ*** by performing the nonlinear parameter estimation task implied by Eq (2.4), nor do we attempt to estimate *n*(***x***, *t*) for any *t* < *t*_0_. From single time point data such as this, an identifiability (i.e., uniqueness) issue arises preventing such estimation from succeeding. This procedure is used in sections 5.1 and 5.2 to produce virtual populations.

Note that one issue that is not addressed in the above technique is uncertainty inherent in the noisy data ***g***_*j*_, namely that ***g***_*j*_ being random means the MLE is also random. While Fisher information analysis can be employed to analyze this uncertainty in an off-line frequentist setting, this analysis does not provide a clear method for producing alternates to ${\hat{\theta }}_{j}$, since one only has access to a single realization of ***g***_*j*_. The Bayesian methodology discussed below provides an avenue towards producing many plausible estimates from a single realization of ***g***_*j*_.

### 4.2. Patient-specific Bayesian solution

In a patient-specific Bayesian solution to the inverse problem $g_{j}=Hf_{j}+\eta_{j}$, we assume that ***f***_*j*_ were sampled from a prior distribution $P_{0}(f)$. Note that $P_{0}(f)$ need not correspond to a well-calibrated model of the true population in order to proceed with the inference, but the method is typically much more reliable if it does. Then, since ***g**_j_* is statistically coupled to ***f**_j_* through Eq (3.3), we can consider the conditional random vector ***g***_*j*_∣***f***_*j*_, whose statistics are described by the likelihood function $L(f_{j}|g_{j})=P(g_{j}|f_{j})$ as discussed previously. A Bayesian inverse solution to the inference problem is to consider the posterior random vector ***f***_*j*_∣***g**_j_* instead of a single point estimate ${\hat{f}}_{j}$. Samples from the posterior can be used to generate a virtual population as described in the procedure above, where now ${\hat{n}}_{j}$ and ${\hat{\rho }}_{j}$ are replaced by samples from their corresponding posteriors to account for uncertainty in the data ***g***_*j*_.

Bayesian personalization of tumor growth has been considered in [21, 54], for example. The former considers an RDE similar to Eq (2.1) but requires two segmented MRI images to perform the personalization. The latter generates ensemble members (i.e., virtual patients) by using fixed RDE coefficients (***β*** in our notation) and imposing an ad hoc additive Gaussian noise model on the system evolution, in order to leverage the ensemble Kalman filter technique.

The distribution of the posterior ***f**_j_*∣***g***_*j*_ is given via Bayes rule, which states informally that $P_{post}(f_{\;j}|g_{\;j})\propto P_{0}(f)L(f_{\;j}|g_{\;j})$. More rigorously [55], we would say that

$$\frac{dP_{post}}{dP_{0}}\propto L(f_{j}|g_{j})=\text{exp}\left(-\ell (f_{j}|g_{j})\right)$$

Practically speaking, in this paper we consider (via Eqs (2.7) and (2.8)) finite-dimensional parameterizations of ***f***, so that when coupled with binned-mode ECT data, the result is a finite-dimensional Bayesian inverse problem [56]. Taking as before a lumpy-type parameterization ***f***_*j*_ = **Φ**(***θ***_*j*_), and with the data described by the nonlinear function ${\bar{g}}_{j}=H(\theta_{j})=H\Phi (\theta_{j})$ defined in section 3.2, we now assign a prior on the vector ***θ***_*j*_, which we assume has PDF *p*_0_(***θ***_*j*_). The Poisson likelihood $L(\theta_{j}|g_{j})$ is given in Eq (3.6), leading to a description of the posterior as

(4.6) $$p(\theta_{j}|g_{j})\propto p_{0}(\theta_{j})L(\theta_{j}|g_{j})$$

If either the prior is infinite-dimensional or the ECT data is assumed to be of particle-processing form, a fully infinite-dimensional Bayesian approach must be considered [55].

To draw samples from the posterior (4.6), we apply a Markov Chain Monte Carlo (MCMC) algorithm based on the one presented in [57]. First, we assume for each tracer ***f***_*j*_ an expansion of the form Eq (2.8), so that ***f***_*j*_ = **Φ**(***θ***_*j*_) with ***θ***_*j*_ = [*b*_1_, … , *b_L_max__*, ***x***_1_, … , ***x***_*L_max_*_, ***γ***_1_, … ***γ***_*L*_*max*__]. Note that in some cases, we reduce the computational burden by fixing the ***x***_*l*_ and/or ***γ***_*l*_ and only sample the remaining parameters. The MCMC procedure is then as follows:

- Given the current sample $\theta_{j}^{\left(n\right)}$, we propose a new sample $\theta_{j}^{\prime }∼\pi \left(\theta_{j}^{\prime }|\theta_{j}^{\left(n\right)}\right)$ by randomly selecting *L*_*mcmc*_ lumps and perturbing their amplitudes *b*_*l*_, location ***x***_*l*_ and shape ***γ***_*l*_ by a Gaussian with diagonal covariance Σ. This proposal is symmetric.
- From $\theta_{j}^{\prime }$, the activity $f_{j}^{\prime }$ is synthesized using Eq (2.8) and the proposed mean image ${\bar{g}}^{\prime }=Hf_{j}^{\prime }$ is simulated using Eq (3.5).
- The acceptance probability is computed using the Metropolis-Hastings rule [25]:
  $$Q(\theta_{j}^{\prime },\theta_{j}^{\left(n\right)})=\text{min}\left\{1,\frac{p(\theta_{j}^{\prime }|g_{j})}{p(\theta_{j}^{\left(n\right)}|g_{j})}\right\}=\text{min}\left\{1,\frac{p_{0}\left(\theta_{j}^{\prime }\right)L\left(\theta_{j}^{\prime }|g_{j}\right)}{p_{0}\left(\theta_{j}^{\left(n\right)}\right)L\left(\theta_{j}^{\left(n\right)}|g_{j}\right)}\right\},$$
  after which we accept $\theta_{j}^{\left(n+1\right)}=\theta_{j}^{\prime }$ with probability $Q(\theta_{j}^{\prime },\theta_{j}^{\left(n\right)})$, and take $\theta_{j}^{\left(n+1\right)}=\theta_{j}^{\left(n\right)}$ with probability $1-Q(\theta_{j}^{\prime },\theta_{j}^{\left(n\right)})$.

By construction of the Metropolis-Hastings algorithm, the Markov chain samples $\theta_{j}^{\left(1\right)}$, $\theta_{j}^{\left(2\right)}$, … have stationary distribution given by the posterior *p*(***θ***_*j*_∣***g***_*j*_), and can thus be used to synthesize virtual patients for the calculation of any quantitative biomarkers as discussed in section 2.4. An example of this approach is discussed in section 5.1. Note that the standard Metropolis-Hastings algorithm performs best for ***θ*** with relatively small dimension; various modifications for high-dimensional problems have also been proposed [25, 30, 55].

### 4.3. Calibration of population distributions

As illustrated in Figure 2, the population parameter ***θ***_*p*_ which specifies the random field models used to generate the virtual population has a substantial influence on the statistics of any biomarkers calculated using Eq (2.11). The question of how to estimate ***θ***_*p*_ from data thus arises. Such calibration problems in the context of VCTs have been discussed in [58, 59], but their methodology accounts only for ODE models arising in systems pharmacology and does not directly apply to our context of PDE tumor growth models, random fields and imaging data. In this section, we discuss briefly a technique to estimate the population parameters which specify a lumpy-type model (2.8) from a database of ECT images ***G*** = [***g***_1_, … , ***g***_*J*_].

Suppose we assume a lumpy-type model for each of the RDE coefficient fields, ***β*** = [***D*, *ρ*, *κ***], say each is of the form given in Eq (2.8) with *ℓ*(***x***) given by a Gaussian (2.9). While the lump function *ℓ*(***x***) can itself be the target of calibration (see [60], for instance), we assume Eq (2.9) for simplicity. The statistics of the random field $LB(\bar{L},b_{0},\sigma^{2})$ are thus fully specified by the three scalars $\bar{L}$, *b*_0_ and *σ*^2^, which we write as $\theta_{p}=[\bar{L},b,\sigma^{2}]\in \Theta =(0,\infty {)}^{3}$. The problem of calibrating a population distribution such as this is to take a database of noisy image data ***G*** = [***g***_1_, … , ***g***_*J*_] for a population of *J* patients and produce an estimate ${\hat{\theta }}_{p}=\left[\hat{\bar{L}},\hat{b},\hat{\sigma^{2}}\right]$. We can again apply either MLE or the Bayes approach as discussed in sections 4.1 and 4.2.

To develop a likelihood model for ***θ***_*p*_, we consider first the generic case where ***g***_*j*_ consists of direct images of some activity ***f***_*j*_, for instance ***f***_*j*_ ∝ ***ρ***_*j*_ as discussed in section 5.1. This case corresponds to that considered in [61]. Assuming inter-subject independence, we have

(4.7) $$L(\theta_{p}|G)=p(G|\theta_{p})=\prod_{j=1}^{J}P(g_{j}|\theta_{p})$$

Using the law of total probability and the fact that the distribution of the image data is independent of the object statistics, we can write

(4.8) $$P(g_{j}|\theta_{p})=E_{f_{j}|\theta_{p}}\left[P(g_{j}|f_{j})\right]$$

In (binned-mode) ECT imaging, the probability distribution *P*(***g***_*j*_∣***f***_*j*_) is given by the Poisson (3.4), which depends on the imaging operator H. The expression (4.8) states that the probability of observing a given image ***g***_*j*_ if the population parameter is ***θ***_*p*_ is the probability of observing ***g***_*j*_ given the object ***f***_*j*_, averaged over of all possible random field realizations ***f***_*j*_ with statistics specified by the parameter ***θ***_*p*_. The expectation in Eq (4.8) is ostensibly infinite dimensional (since the ***f***_*j*_ are functions), though it can be approximated using Monte Carlo:

(4.9) $$P(g_{j}|\theta_{p})\approx \frac{1}{J^{\prime }}\sum_{j^{\prime }=1}^{J^{\prime }}P(g_{j}|f_{j^{\prime }}),\;\;f_{j^{\prime }}∼P(f_{j}|\theta_{p})$$

Equation (4.9) specifies a computational method for approximating the likelihood (4.7): For fixed ***θ***_*p*_, compute Eq (4.9) for each image in the database, then compute the product (or its logarithm) in Eq (4.7). The MLE of ***θ***_*p*_ is then defined as before in Eq (4.3), with ***θ***_*p*_ ∈ **Θ**_*p*_, where **Θ**_*p*_ is the set of admissible population parameters. Note that the estimate (4.9) can be computed in parallel using graphics processors, which drastically reduces the time required for each likelihood calculation, which would have previously been a bottleneck in the application of a traditional MLE. Despite this, the MLE optimization problem (4.3) applied to the likelihood (4.7) poses a challenge for standard numerical optimization routines due to scaling and non-convexity issues. See section 6 for a brief discussion of future work aimed at addressing these issues.

## 5. Results

In this section, we discuss two in silico experiments that illustrate the methodologies discussed above. For both, a common ‘ground truth’ cell density path *n*(***x***, *t*) is generated by sampling a random field for each coefficient and simulating until *t* = *T* = 365 days. In the first experiment, we use (simulated) ECT data for *f*_*j*_(***x***) = *n*(***x***, 100) to compute both an MLE and a collection of posterior samples for the tumor cell density at *t* = *t*_0_ = 100 days. Then, a virtual population is generated by using the resulting ${\hat{n}}_{j}$ (or posterior samples) as the initial condition in Eq (2.1), randomizing the remaining coefficients and simulating until *t* = *T*. The resulting populations are then compared to the ‘true’ *n*(***x***, *t*), using the tumor burden *N*(*t*) defined in Eq (2.13) as the quantity-of-interest. In the second experiment, we use ECT data and MLE for both the growth factor *ρ*(***x***) and the cell density *n*(***x***, *t*_0_), again generating a VPP by using the estimated cell density as the initial condition *n*_0_(***x***) and $\rho_{j}={\hat{\rho }}_{j}$, then randomizing both ***D*** and ***κ***.

**Figure 4.**
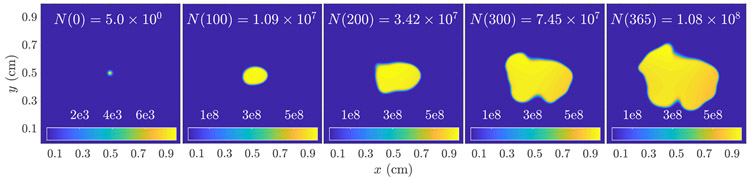
The ‘true’ tumor cell density, generated using Eq (2.1) with ***β*** = (***D*, *ρ*, *κ***) sampled from the random field models specified in the section 5 introduction above. The simulated imaging studies are performed at *t* = 100 days (second panel).

For both experiments, we use the following random fields, both to generate the initial ‘true’ tumor path *n*(***x***, *t*) and to randomize unmeasured coefficients. We assume ***D*** ~ *LB*(20, 1e−7, 0.04), ***ρ*** ~ *LB*(200, 0.25, 0.002), and ***κ*** ~ *LB*(100, 5e7, 0.1). These parameters were selected to achieve an average tumor size of approximately 10^8^ cells after the first year; obviously a calibration procedure such as the one outlined in section 4.3 would be required to estimate population parameters for a real population of tumors. The exact sample used is shown in Figure 4.

### 5.1. Experiment 1: Maximum likelihood estimation and posterior sampling of cell density n

In this experiment, we suppose that $g_{j}^{\left(n\right)}=H^{\left(n\right)}n_{j}+\eta_{j}^{\left(n\right)}$ consists of ECT measurements of the cell density *n*_*j*_(***x***, *t*_0_), with *t*_0_ = 100 days, and that no other measurements are available. We assume that the experimental setup is confined to a thin slab so that a 2D simulation described in section 3.1 is appropriate, and that the imaging system corresponds to Eq (3.1) with blur kernel given by Eq (3.2). The blur is taken to be $\sigma_{blur}^{2}=0.0212$, which corresponds to an imaging resolution (Full-Width-Half-Max, FWHM) of 500 *μ*m, and we assume that the photon yield is *A* = 1e−3 (detected photons per cell). Once the mean image data ${\bar{g}}_{j}=Hf_{j}$ is computed using Eq (3.1), a randomized image is produced using poissrnd.

**Figure 5.**
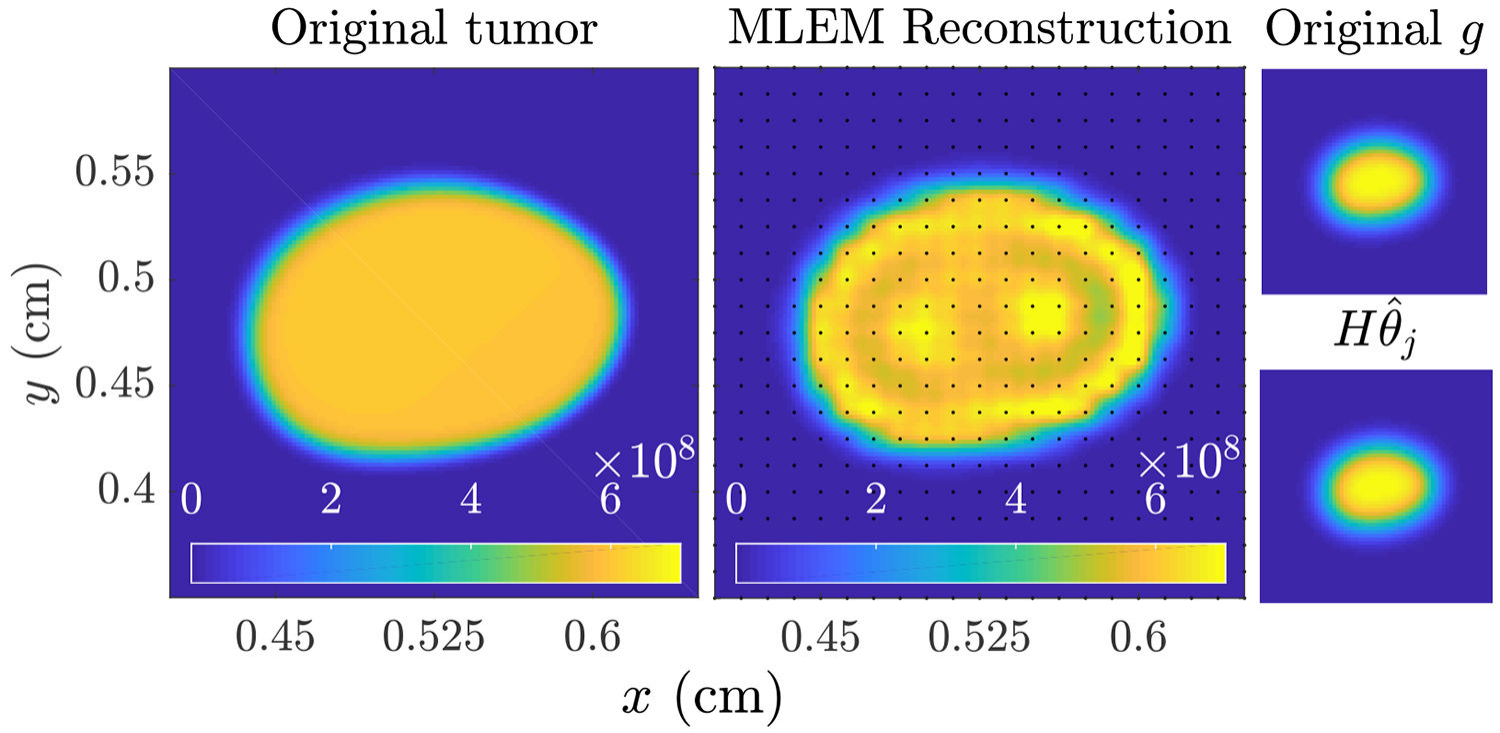
Comparison of the ‘true’ tumor shown in Figure 4 (left, imaged at *t*_0_ = 100 days) to the maximum likelihood reconstruction ${\hat{n}}_{j}^{\left(ML\right)}$ (middle). The original noisy image $g_{j}=Hn_{j}+\eta_{j}$ and the mean image evaluated at the MLE ${\bar{g}}_{j}=H{\hat{n}}_{j}^{\left(ML\right)}=H{\hat{\theta }}_{j}^{\left(ML\right)}$ are shown to the right. The MLEM algorithm (4.4) with 5000 iterations was employed. The reconstruction grid is shown on the middle plot (black dots). The computational time required for this reconstruction was about 30 seconds.

To perform the image reconstruction for *n*_*j*_(***x***, *t*_0_), we apply the linear MLEM algorithm (4.4) with a reconstruction of the type (2.8), with fixed lump centers and lump variance, so that ***θ*** = [*b*_1_, … , *b*_*L_max_*_]. Specifically, we fix the lump centers on a grid of size 25 × 25, uniformly spaced on [0.35, 0.65]^2^, and set the lump variance to *σ*^2^ = 1e−2/256. The matrix ***H*** with columns given by ${\bar{g}}_{l}=H(\ell (x-x_{l}))$ is then computed using Eq (3.1), and the MLEM algorithm (4.4) applied for *K*_*max*_ = 5000 iterations, with initial guess ${\hat{\theta }}_{j}^{\left(0\right)}=H^{T}g_{j}$. From the final ${\hat{\theta }}_{j}^{\left(K_{max}\right)}$, we synthesize the estimate ${\hat{n}}_{j}(x,t_{0})$ using Eq (2.8). The results of this reconstruction are shown in Figure 5.

**Figure 6.**
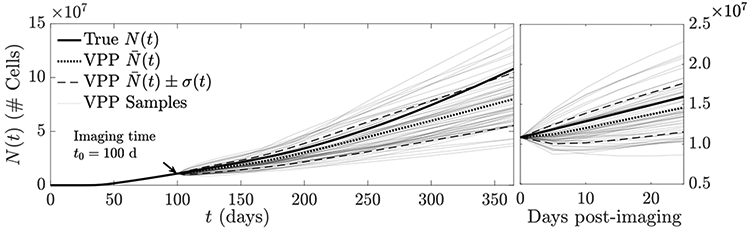
Illustration of a VPP-based prediction of tumor burden *N*(*t*) when an MLE of *n*(***x***, *t*_0_) is used as the initial condition and remaining coefficients are randomized (*J* = 64). The ‘true’ tumor path (bold), empirical average of the VPP (dotted) and a one standard deviation confidence band (dashed line) are shown. Individual VPP samples are shown in light grey. The left plot shows the time interval from 0 (tumor initiation) to 1 year, while the right shows the interval from imaging to 30 days post-imaging. The VPP appears biased towards slower trajectories than the actual.

**Figure 7.**
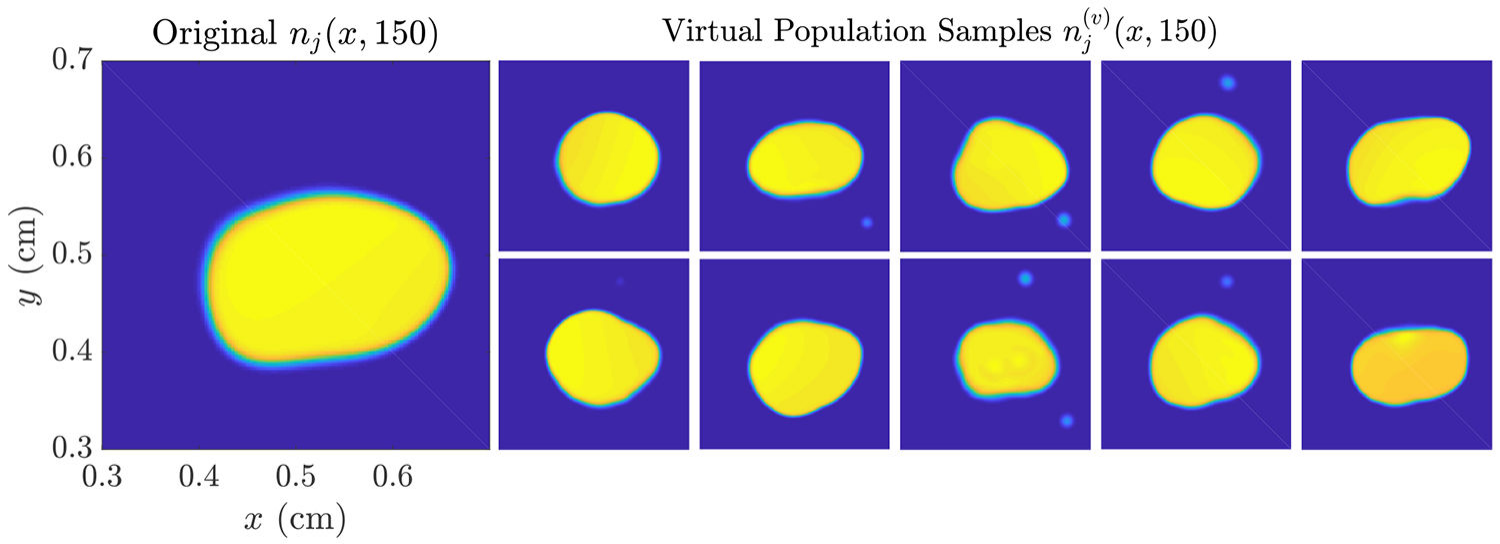
Example VPP Samples for *t* = 150, i.e., 50 days post-imaging, generated using the MLEM estimate of *n*_*j*_(***x***, 100) as the initial condition and randomizing the other coefficient fields as described in the main text. All plots are shown on [0.3, 0.7]^2^ and with a common colorbar. It is apparent that the tumor morphology is largely consistient across the population, though some virtual tumors demonstrate small ‘islands’ of growth and the overall rate of growth is variable, as seen in Figure 6.

To demonstrate the usage of this estimate to generate a virtual tumor population as discussed in section 4.1, we use the resulting ${\hat{n}}_{j}(x,t_{0})$ as the initial condition *n*_0_(***x***) in Eq (2.1), and randomize the remaining coefficient fields using the lumpy-type random field models described in the introduction to this section to produce a virtual population of size *J* = 64. In Figure 6, the quantity-of-interest *N*(*t*) is shown for the resulting virtual population, while in Figure 7, a collection of 10 virtual tumors is shown for *t* = 150 (i.e., 50 days post-imaging).

**Figure 8.**
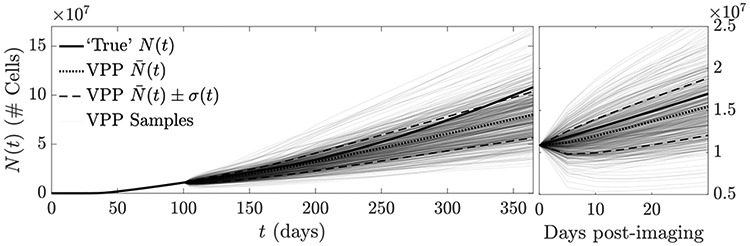
Illustration of the virtual population generated using Bayesian posterior sampling for the initial condition ***n***_0_(***x***). The empirical average path $\bar{N}(t)$(dotted) and confidence band (dashed) are shown in comparison to the true tumor path (solid), as well as the *J* = 512 paths constituting the virtual population (light grey).

To demonstrate the usage of the Bayesian methods discussed in section 4.2, we generated samples from the posterior ***n***_*j*_∣***g***_*j*_ to use as part of the virtual population generation procedure. For the MCMC procedure, we use the algorithm outlined in section 4.2, moving *L*_*mcmc*_ = 3 lumps for each proposal with a proposal distribution of $N(0,\sigma_{mcmc}^{2}I)$, taking *σ*_*mcmc*_ = 1e4. The prior for ***θ***_*j*_ = [*b*_1_, … , *B_L_max__*] is chosen to be I.I.D. uniform on the interval [0, 1e10]. Starting from $\theta_{j}^{\left(0\right)}={\hat{\theta }}_{j}^{\left(ML\right)}$, i.e., starting at the maximum likelihood estimate, we generate *J* = 512 samples, then synthesize $n_{j}^{\left(1\right)},\ldots ,n_{j}^{\left(J\right)}$. Note that the subscript remains *j* as these samples correspond to patient *j*, while the superscript indicates that these are posterior samples, generated using MCMC. Starting at the maximum likelihood estimate (which for our prior corresponds to the MAP estimate) reduces the need for an expensive burn-in period in which the first *J*_0_ samples are thrown out to ensure that the chain is sampling from the posterior. The acceptance rate for our chain (number of proposals accepted divided by total number of samples) is 0.495. Using each posterior sample as an initial condition, we again generate a virtual population by solving Eq (2.1) with the remaining coefficients ***D*, *ρ*, *κ*** randomized according to the lumpy-type field models specified previously. The results of this virtual population are shown in Figure 8.

For both the maximum likelihood and Bayesian solutions, the resulting virtual populations underestimate, on average, the true tumor burden both over the short- and long-term, which indicates that additional data may be needed to estimate other model coefficients to gain a more accurate patient-specific prediction; we discuss on possibility in section 5.2 below. While the Bayesian technique accounts for uncertainty inherent in the image data $g_{j}^{\left(n\right)}$, this uncertainty appears relatively small as it relates to the prediction of *N*(*t*); the variance of *N*(*t*) for the virtual population generated using the Bayesian technique is only slightly larger than the variance for the MLE population.

### 5.2. Experiment 2: Maximum likelihood estimation of both n and ρ

**Figure 9.**
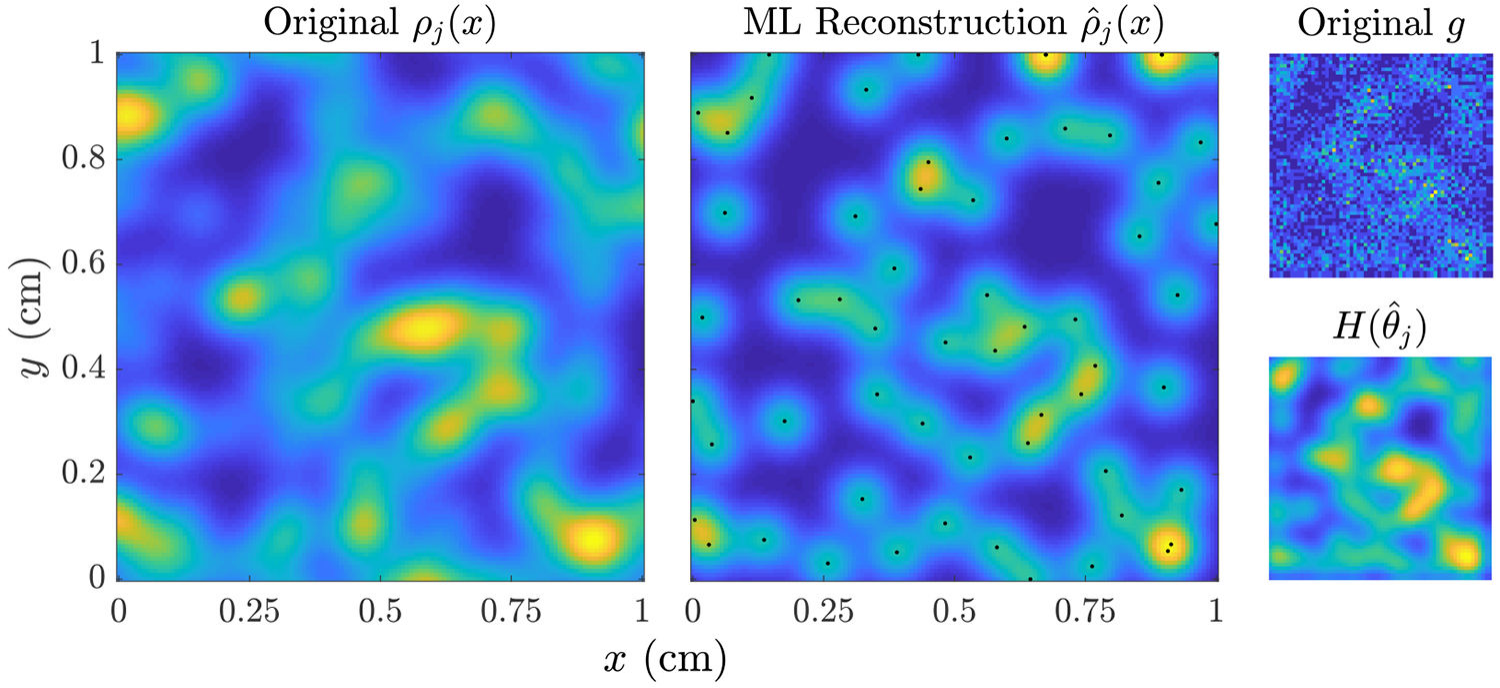
Maximum likelihood reconstruction of the growth factor ***ρ***_*j*_ (left) from simulated imaging data (top right). The reconstruction (middle) with the resulting lump centers highlighted (black dots) are shown, as well as the mean image at the MLE ${\bar{g}}_{j}^{\rho }=H^{\left(\rho \right)}{\hat{\rho }}_{j}$ (bottom right). The computational time for this reconstruction was several hours. While the reconstruction may appear visually poor, it is sufficient for the task of predicting *n*(***x***, *t*) and *N*(*t*), as illustrated in Figures 10 and 11.

In Experiment 1, we saw that the virtual population under-estimated the true tumor path in both the MLE and Bayesian examples, suggesting that additional data may be required. In this experiment, we assume that the image data ***g***_*j*_ consists of (simulated) polyscopic data of the following form. We assume that we have access first to cell density measurements of the form $g_{j}^{\left(n\right)}=H^{\left(n\right)}n_{j}+\eta_{j}^{\left(n\right)}$, where $H^{\left(n\right)}$ is the same as in Experiment 1. The second dataset is of the form $g_{j}^{\left(\rho \right)}=H^{\left(\rho \right)}\rho_{j}+\eta_{j}^{\left(\rho \right)}$, where we now have $\sigma_{blur}^{2}=0.0425$ (1 mm FWHM). As in the first experiment, we assume that imaging is performed at *t* = *t*_0_ = 100. The image reconstruction for ***n***_*j*_ is performed using MLEM as in experiment 1 (Figure 5). For *ρ*_*j*_(***x***), we use MLE with a nonlinear synthesis map as follows. The lumpy-type expansion (2.8) with Gaussian lump (2.9) is assumed where now ***θ***_*j*_ = [***x***_1_, … , ***x***_*L_max_*_, *b*, *σ*^2^], that is, we fit the lump positions and a common lump amplitude and variance. The result is a reconstruction of the form

$${\hat{\rho }}_{j}=\hat{b}\sum_{l=1}^{L_{max}}\text{exp}\left(-\frac{1}{2{\hat{\sigma }}^{2}}\|x-{\hat{x}}_{l}{\|}^{2}\right),$$

where we have chosen *L*_*max*_ = 60 for this experiment. We assume further the constraints that ***x***_*l*_ ∈ [0, 1]^2^, *b* ∈ (0, ∞), and *σ*^2^ ∈ (0, 0.1], and use the Matlab algorithm fmincon to perform the MLE optimization (4.3). The results of this reconstruction are shown in Figure 9.

**Figure 10.**
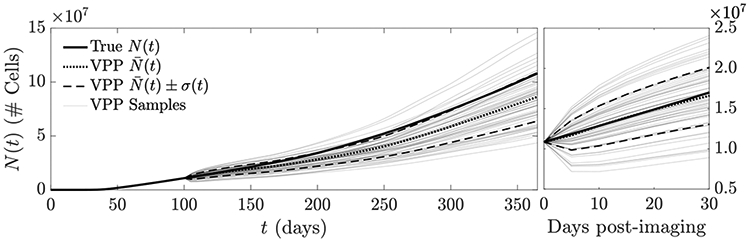
Prediction of tumor burden *N*(*t*) when MLEs of *n*(***x***, *t*_0_) and *ρ*(***x***) are used for the initial condition and growth factor. *D*(***x***) and *κ*(***x***) are randomized to produce a VPP (*J* = 64). The original tumor path (bold) and empirical average of the VPP (dotted), as well as a one-*σ* confidence band (dashed) and individual VPP samples (light grey) are shown. The left plot shows *t* = 0 to *t* = 365, while the right plot shows *t* = 100 (imaging time) to *t* = 130. Over the 30 day post-imaging window, the VPP appears nearly unbiased (compare to Figure 6).

**Figure 11.**
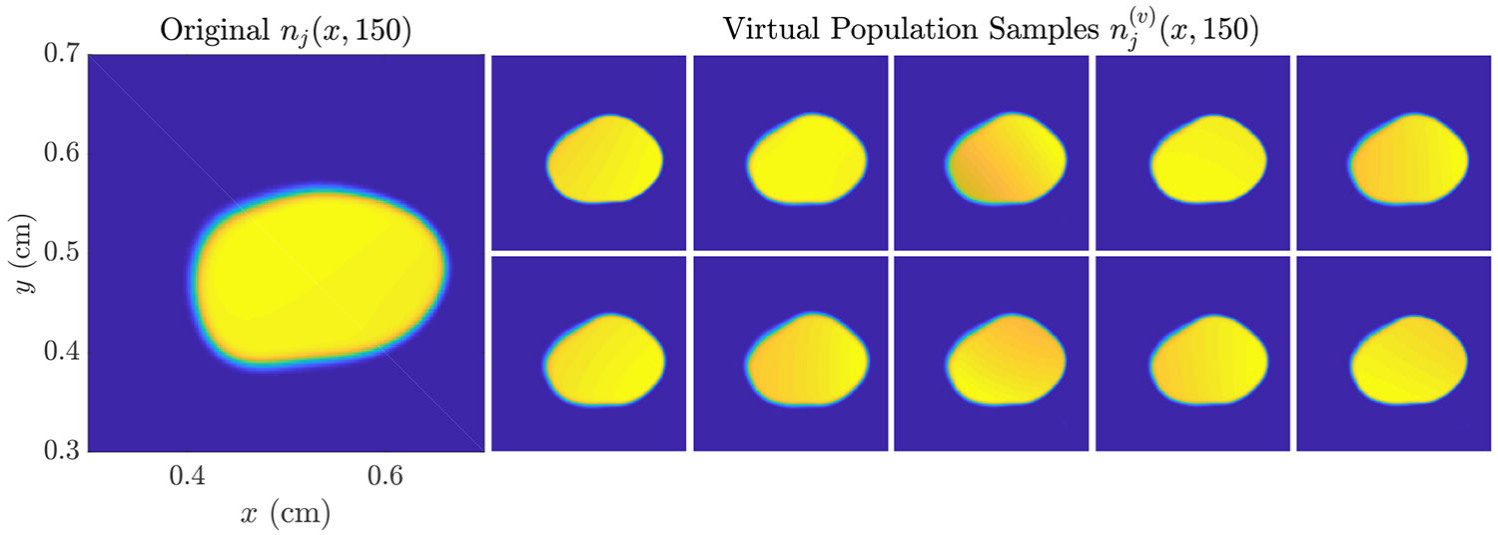
Example VCT Samples for *t* = 150, i.e. 50 days post-imaging, generated using the MLEM estimate of *n*_*j*_(***x***, 100) as the initial condition, the MLE of *ρ*_*j*_(***x***) for the growth factor, and randomizing *D*(***x***) and *κ*(***x***) as described in the main text. All plots are shown on [0.3, 0.7]^2^ and with a common colorbar. In contrast to Figure 7, the morphology apparent in the virtual population is nearly identical; heterogeneity in the overall growth is apparent in Figure 10.

In Figure 10, we compare the tumor burden predicted using the virtual population to the ‘true’ tumor path. Over the first 30 day post-imaging period, the ensemble mean $\bar{N}(t)$ for the virtual population tracks the true tumor path closely, indicating that if both ***n*** and ***ρ*** are estimated, the result appears unbiased over this short time period. This is also demonstrated by looking at the individual virtual population samples in Figure 11: Visual inspection reveals a closer overall appearance to the ‘true’ tumor than was apparent in Figure 7. Over a longer time period, the ensemble still trends towards a lower overall growth rate than the ‘true’ tumor path, as seen previously in Figure 6.

## 6. Discussions

In this article, we have presented a rigorous, end-to-end statistical framework for addressing both inter- and intra-patient heterogeneity for the commonly used reaction-diffusion tumor growth model (2.1), emphasizing the usage of raw molecular emission imaging data to estimate unknown model parameters. We demonstrated these methods in two in silico experiments, using a simulated ground truth tumor path and simulated imaging data to illustrate patient-specific Virtual Patient Population (VPP) generation.

In section 2.3, we presented a family of random field models that are both easy to simulate and demonstrate a wide variety of spatial heterogeneity; in section 4.3, we discussed how the parameters defining the statistics of these random field models can be estimated from a collection of molecular images, and discussed some of the computational challenges inherent in this task. Future work will discuss algorithms for solving this MLE problem, as well as additional estimation strategies to calibrate more general random field models from noisy data.

In section 3, we discussed molecular Emission Computed Tomography (ECT) imaging systems and their associated statistical models. For simplicity, we emphasized the case of performing direct, possibly polyscopic, 2D imaging of a thin sample. While the 2D approximation is very limiting in general, it is applicable to the experimental setting of mouse window chambers, a topic of future work in our group. In another future work, we will address fully 3D tomographic ECT systems with particle-processing data and the associated computational and image quality questions that arise. Understanding image data quality in a task performance setting can help design better imaging systems [51], and to our knowledge, there is currently minimal effort formally evaluating the quality of image data in the mathematical oncology context.

In section 4, we presented general likelihood-based statistical procedures for estimating both patient-specific and population parameters from noisy molecular imaging data, and in section 5, we presented two experiments to illustrate these procedures. In the first experiment, measurements of *n*_*j*_(***x***, 100) are used to construct a maximum likelihood estimate and posterior samples, which are subsequently used to generate a patient-specific VPP. In the second experiment, we add an additional imaging study, using only maximum likelihood to estimate both *n*_*j*_(***x***, 100) and *ρ*_*j*_(***x***). The resulting virtual population demonstrated a more accurate prediction of the true *N*_*j*_(*t*), over a 30 day post-imaging prediction period. In a future work, we will investigate the question of optimizing imaging acquisition schemes for the task of predicting *N*(*t*). We will also address the addition of treatment models and evaluation techniques discussed in [5] to our virtual population methodology, working towards providing a general strategy to solving the optimization problem (2.12).
